# Supplementary material for: INHABIT: A web-based decision support tool for invasive plant species habitat visualization and assessment across the contiguous United States
Source: PLoS One. 2022 Feb 8;17(2):e0263056. doi: 10.1371/journal.pone.0263056 (PMC8824347; doi:10.1371/journal.pone.0263056)
Supplement: S1 Table — The variable column includes the name, listed within brackets are the unit of measurement, spatial (cell) resolution, and temporal resolution (if applicable). Each variable also has a specific description and source. Red variable names represent first version variables removed from the second version of the predictor list due to high correlations, redundancy, or contributing little importance to models. Yellow variable names represent second version variables that replaced a similar predictor from the first version. Green variable names represent second version variables that were created in response to feedback and added to the second version of the predictor list to improve model sensitivity. Variable names without an associated color represent first version variables that were retained in the second version list without being modified. (DOCX) [file pone.0263056.s001.docx]

**SUPPLEMENTARY MATERIAL**

**S1 Table.** **List of environmental variables considered for invasive habitat suitability modeling.**

| **Variable** | **Description** | **Source** | **Notes** |
| --- | --- | --- | --- |
| Annual Mean Temperature (bio1)  [°C \| 800m \| 1981 – 2010] | Annual mean temperature has climate inputs that consist of monthly mean maximum and minimum temperatures, which are averaged across the year to acquire annual mean temperature. The annual mean temperature approximates the total energy inputs for an ecosystem. | [PRISM](http://www.prism.oregonstate.edu) & O’Donnell and Ignizio 2012 ^1^ | High correlations and redundancy |
| Mean Diurnal Range (bio2)  [°C \| 800m \| 1981 – 2010] | Mean diurnal range is the mean of all the averaged, monthly temperature extremes whose inputs are monthly mean maximum and minimum temperatures. It is calculated by finding the difference between the maximum and minimum temperature for each month, and then averaging these values, which can inform the relevance of temperature fluctuation to different species. | [PRISM](http://www.prism.oregonstate.edu) & O’Donnell and Ignizio 2012 ^1^ |  |
| Isothermality (bio3)  [percent \| 800m \| 1981 – 2010] | Isothermality quantifies how much the day-to-night temperatures oscillate relative to the summer-to-winter oscillations. Inputs include monthly mean maximum and monthly mean minimum temperatures. | [PRISM](http://www.prism.oregonstate.edu) & O’Donnell and Ignizio 2012 ^1^ |  |
| Temperature Seasonality (bio4)  [std dev * 100 \| 800m \| 1981 – 2010] | The amount of temperature variation over the given period based on the standard deviation of monthly temperature averages. This is calculated by averaging the minimum temperature and maximum temperature for each month and then calculating the standard deviation of the 12 mean monthly temperature values. | [PRISM](http://www.prism.oregonstate.edu) & O’Donnell and Ignizio 2012 ^1^ | High correlations and redundancy |
| Temperature Seasonality (bio4a)  [std dev * 100 \| 800m \| 1981 – 2010] | The amount of temperature variation over the given period based on the standard deviation of average monthly temperatures. Values are first converted to degrees Kelvin to avoid negative temperature and the possibility of having to divide by zero. It is calculated by averaging the minimum temperature and maximum temperature for each month and then calculating the standard deviation of the 12 mean monthly temperature values. | [PRISM](http://www.prism.oregonstate.edu) & O’Donnell and Ignizio 2012 ^1^ | High correlations and redundancy |
| Maximum Temperature Warmest Month (bio5)  [°C \| 800m \| 1981 – 2010] | Maximum temperature of warmest month is the monthly mean maximum temperature occurrence over a given period. This can inform whether species distributions are affected by warm anomalies throughout the year. | [PRISM](http://www.prism.oregonstate.edu) & O’Donnell and Ignizio 2012 ^1^ | High correlations and redundancy |
| Minimum Temperature Coldest Month (bio6)  [°C \| 800m \| 1981 – 2010] | Minimum temperature of coldest month is the monthly mean minimum temperature occurrence over a given period. This can inform whether species distributions are affected by cold anomalies throughout the year. | [PRISM](http://www.prism.oregonstate.edu) & O’Donnell and Ignizio 2012 ^1^ | High correlations and redundancy |
| Temperature Annual Range (bio7)  [°C \| 800m \| 1981 – 2010] | Temperature annual range is a measure of temperature variation over a given period calculated by subtracting minimum temperature of coldest month (BIO 6) from maximum temperature of warmest month (BIO5). This can inform whether species distributions are affected by ranges of extreme climatic conditions. | [PRISM](http://www.prism.oregonstate.edu) & O’Donnell and Ignizio 2012 ^1^ |  |
| Mean Temperature of Wettest Quarter (bio8)  [°C \| 800m \| 1981 – 2010] | Approximate mean temperatures that prevail during the wettest season. It is calculated by identifying the three consecutive months with the highest cumulative precipitation total; The maximum and minimum temperature for each of the 3 selected months is averaged, and the monthly averages are averaged. | [PRISM](http://www.prism.oregonstate.edu) & O’Donnell and Ignizio 2012 ^1^ | High correlations and redundancy |
| Mean Temperature of Driest Quarter (bio9)  [°C \| 800m \| 1981 – 2010] | Approximate mean temperatures that prevail during the driest season. It is calculated by identifying the three consecutive months with the lowest cumulative precipitation total; The maximum and minimum temperature for each of the 3 selected months is averaged, and the monthly averages are averaged. | [PRISM](http://www.prism.oregonstate.edu) & O’Donnell and Ignizio 2012 ^1^ | High correlations and redundancy |
| Mean Temperature of Warmest Quarter (bio10)  [°C \| 800m \| 1981 – 2010] | Approximate mean temperatures that prevail during the warmest season, calculated by identifying the warmest quarter of the year (the average temperatures of each month in the quarter are summed). The maximum and minimum temperatures are averaged for each of the 3 selected months, and the monthly averages are averaged. | [PRISM](http://www.prism.oregonstate.edu) & O’Donnell and Ignizio 2012 ^1^ | High correlations and redundancy |
| Mean Temperature of Coldest Quarter (bio11)  [°C \| 800m \| 1981 – 2010] | Approximate mean temperatures that prevail during the coldest season, calculated by identifying the coldest quarter of the year (the average temperatures of each month in the quarter are summed). The maximum and minimum temperatures are averaged for each of the 3 selected months, and the monthly averages are averaged. | [PRISM](http://www.prism.oregonstate.edu) & O’Donnell and Ignizio 2012 ^1^ | High correlations and redundancy |
| Annual Precipitation (bio12)  [mm \| 800m \| 1981 – 2010] | The sum of all total monthly precipitation estimates, which approximates the total water inputs. This can inform the importance of water availability to species distributions. | [PRISM](http://www.prism.oregonstate.edu) & O’Donnell and Ignizio 2012 ^1^ | High correlations and redundancy |
| Precipitation of Wettest Month (bio13)  [mm \| 800m \| 1981 – 2010] | The total precipitation that prevails during the wettest month. Extreme precipitation conditions during the year influences a species' potential range. If is derived from climate normals, then the wettest month is based on a span of years. | [PRISM](http://www.prism.oregonstate.edu) & O’Donnell and Ignizio 2012 ^1^ | High correlations and redundancy |
| Precipitation of Driest Month (bio14)  [mm \| 800m \| 1981 – 2010] | The total precipitation that prevails during the driest month Extreme precipitation conditions during the year influences a species' potential range. If it is derived from climate normals, then the driest month is based on a span of years. | [PRISM](http://www.prism.oregonstate.edu) & O’Donnell and Ignizio 2012 ^1^ | High correlations and redundancy |
| Precipitation Seasonality (Cv) (bio15)  [percent \| 800m \| 1981 – 2010] | A measure of the variation in monthly precipitation totals over the course of the year. This index is the ratio of the standard deviation of the monthly total precipitation to the mean monthly total precipitation (aka coefficient of variation) and is expressed as a percentage | [PRISM](http://www.prism.oregonstate.edu) & O’Donnell and Ignizio 2012 ^1^ |  |
| Precipitation of Wettest Quarter (bio16)  [mm \| 800m \| 1981 – 2010] | Approximate total precipitation that prevails during the wettest season. The three consecutive months with the highest cumulative precipitation total are identified, and then the precipitation values are summed for all three months. | [PRISM](http://www.prism.oregonstate.edu) & O’Donnell and Ignizio 2012 ^1^ |  |
| Precipitation of Driest Quarter (bio17)  [mm \| 800m \| 1981 – 2010] | Approximate total precipitation that prevails during the driest season. The three consecutive months with the lowest cumulative precipitation total are identified, and then the precipitation values are summed for all three months. | [PRISM](http://www.prism.oregonstate.edu) & O’Donnell and Ignizio 2012 ^1^ |  |
| Precipitation of Warmest Quarter (bio18)  [mm \| 800m \| 1981 – 2010] | Approximate total precipitation that prevails during the warmest season, calculated by identifying the warmest quarter of the year (the average temperatures of each month in the quarter are summed; the quarter with the highest value is selected). The precipitation values for the three months in this quarter are then summed. | [PRISM](http://www.prism.oregonstate.edu) & O’Donnell and Ignizio 2012 ^1^ |  |
| Precipitation of Coldest Quarter (bio19)  [mm \| 800m \| 1981 – 2010] | Approximate total precipitation that prevails during the coldest season, calculated by identifying the coldest quarter of the year (the average temperatures of each month in the quarter are summed; the quarter with the lowest value is selected). The precipitation values for the three months in this quarter are then summed. | [PRISM](http://www.prism.oregonstate.edu) & O’Donnell and Ignizio 2012 ^1^ | High correlations and redundancy |
| Minimum Winter Temperature  [°C \| 4,000m \| 1981 – 2010] | The monthly mean minimum temperature over the winter months (December through February). | [Climate Engine](https://app.climateengine.org/)^2^ | Updated to include additional years of data [1981 – 2018] |
| Mean Spring Temperature  [°C \| 4,000m \| 1981 – 2010] | Mean temperature of spring months (March through June) | [Climate Engine](https://app.climateengine.org/)^2^ | Updated to include additional years of data [1981 – 2018] |
| Maximum Mean Summer Temperature  [°C \| 4,000m \| 1981 – 2010] | Maximum temperature of summer months (June through August) | [Climate Engine](https://app.climateengine.org/)^2^ | Updated to include additional years of data [1981 – 2018] |
| Maximum Summer Temperature  [°C \| 4,000m \| 1981 – 2010] | Mean maximum temperature of summer months (June through August) | [Climate Engine](https://app.climateengine.org/)^2^ | High correlations and redundancy |
| Mean PET Fall  [mm \| 4,000m \| 1981 – 2010] | Potential water deficit of fall months (October through November) calculated by averaging potential evapotranspiration subtracted from precipitation over a given time period. | [Climate Engine](https://app.climateengine.org/)^2^ | Updated to include additional years of data [1981 – 2018] |
| Mean PET Spring  [mm \| 4,000m \| 1981 – 2010] | Potential water deficit of spring months (March through June) calculated by averaging potential evapotranspiration subtracted from precipitation over a given time period. | [Climate Engine](https://app.climateengine.org/)^2^ | Updated to include additional years of data [1981 – 2018] |
| Mean PET Summer  [mm \| 4,000m \| 1981 – 2018] | Potential water deficit of summer months (June through August) calculated by averaging potential evapotranspiration subtracted from precipitation over a given time period. | [Climate Engine](https://app.climateengine.org/)^2^ |  |
| Mean PET Spring  [mm \| 4,000m \| 1981 – 2018] | Potential water deficit of early spring months (March through May) calculated by averaging potential evapotranspiration subtracted from precipitation over a given time period. | [Climate Engine](https://app.climateengine.org/)^2^ |  |
| Mean PET  [mm \| 4,000m \| 1981 – 2010] | Potential water deficit of fall to spring months (October to June) calculated by averaging potential evapotranspiration subtracted from precipitation over a given time period. | [Climate Engine](https://app.climateengine.org/)^2^ | Updated to include additional years of data [1981 – 2018] |
| Mean PET Growing Season  [mm \| 4,000m \| 1981 – 2018] | Potential water deficit of the growing season (April to October) calculated by averaging potential evapotranspiration subtracted from precipitation over a given time period. | [Climate Engine](https://app.climateengine.org/)^2^ |  |
| Mean Precipitation Spring  [mm \| 4,000m \| 1981 – 2018] | Mean precipitation of early spring (March-May) | [Climate Engine](https://app.climateengine.org/)^2^ |  |
| Mean Precipitation Spring  [mm \| 4,000m \| 1981 – 2010] | Mean precipitation of spring months (March through June) | [Climate Engine](https://app.climateengine.org/)^2^ | Updated to include additional years of data [1981 – 2018] |
| Mean Precipitation March  [mm \| 4,000m \| 1981 – 2010] | Mean precipitation of March | [Climate Engine](https://app.climateengine.org/)^2^ | Updated to include additional years of data [1981 – 2018] |
| Mean Precipitation March/Mean Precipitation Spring  [fraction/mm \| 4,000m \|1981 – 2010] | Mean precipitation of March divided by Mean precipitation of spring months (March through June) | [Climate Engine](https://app.climateengine.org/)^2^ | Updated to include additional years of data [1981 – 2018] |
| Mean Precipitation Growing Season  [mm \| 4,000m \| 1981 – 2017] | Mean precipitation of the growing season (April through October) | [Climate Engine](https://app.climateengine.org/)^2^ |  |
| Mean Annual Precipitation  [mm \| 4,000m \| 1981 – 2017] | Mean annual precipitation | [Climate Engine](https://app.climateengine.org/)^2^ |  |
| Evapotranspiration: Oct-Nov  [mm \| 1,000m \| 2003 – 2017] | Mean monthly evapotranspiration of fall months (October through November) | [Climate Engine](https://app.climateengine.org/)^2^ |  |
| Evapotranspiration: Mar-Jun  [mm \| 1,000m \| 2003 – 2017] | Mean monthly evapotranspiration of spring months (March through June) | [Climate Engine](https://app.climateengine.org/)^2^ |  |
| Evapotranspiration: Apr-Oct  [mm \| 1,000m \| 2003 – 2017] | Mean monthly evapotranspiration of the growing season (April through October) | [Climate Engine](https://app.climateengine.org/)^2^ |  |
| Evapotranspiration: Oct-Jun  [mm \| 1,000m \| 2003 – 2017] | Mean monthly evapotranspiration of fall to spring (October through June) | [Climate Engine](https://app.climateengine.org/)^2^ |  |
| Evapotranspiration: Mar-May  [mm \| 1,000m \| 2003 – 2018] | Mean monthly evapotranspiration of spring months (March through May) | [Climate Engine](https://app.climateengine.org/)^2^ |  |
| Evapotranspiration: Jun-Aug  [mm \| 1,000m \| 2003 – 2018] | Mean monthly evapotranspiration of summer months (June through August) | [Climate Engine](https://app.climateengine.org/)^2^ |  |
| Burning Index  [0-100 \| 4,000m \| 1981 – 2010] | Burning index mean (based on the National Fire Danger Rating System) | [Climate Engine](https://app.climateengine.org/)^2^ |  |
| Global Human Modification (gHM)  [0-1 \| 1,000m \| 2016 median] | The global Human Modification map (HM) provides a cumulative measure of human modification of terrestrial lands across the globe. It is a continuous metric that reflects the proportion of a landscape modified based on modeling the physical extents of 13 anthropogenic stressors and their estimated impacts using spatially explicit global datasets. | [gHM](https://figshare.com/articles/Global_Human_Modification/7283087)^3^ | Added to provide a more holistic measure of human modification through a wider variety of sources |
| Landscape Condition Model  [0-1 \| 90m \| 2017] | The landscape condition model identifies human land uses (built infrastructure, agriculture, vegetation alteration, etc.) | [Nature Serve](http://www.natureserve.org/conservation-tools/modeling-landscape-condition)^4^ | Replaced with gHM |
| Human Influence Index  [0-64 \| 1,000m \| 1995 – 2014] | Human influence index identifies anthropogenic impacts on the environment. It incorporates nine global data layers covering human population pressure (population density), human land use and infrastructure (built-up areas, nighttime lights, land use/land cover), and human access (coastlines, roads, railroads, navigable rivers). | [NASA - SEDAC](http://sedac.ciesin.columbia.edu/data/set/wildareas-v2-human-influence-index-geographic)^5^ | Replaced with gHM |
| Mean Annual Flow  [cubic ft/sec \| 90m \| 1971 – 2000] | The mean annual flow rate (cfs) (with gage adjustments) of the flowline features; estimated from each flowline feature's catchment (drainage) area. | [NHDPlus](https://www.sciencebase.gov/catalog/item/56c38ad8e4b0946c6520aa52)^6^ | Added to address INHABIT feedback and improve model sensitivity within riparian areas |
| Percent Clay (0-5cm)  [percent \| 100m] | Mean percent clay cover in the first 0-5cm of the soil horizon | [POLARIS](https://www.sciencedirect.com/science/article/pii/S0016706116301434)^7^ | Replaced with new dataset based on expert feedback |
| Percent Sand (0-5cm)  [percent \| 100m] | Mean percent sand cover in the first 0-5cm of the soil horizon | [POLARIS](https://www.sciencedirect.com/science/article/pii/S0016706116301434)^7^ | Replaced with new dataset based on expert feedback |
| Percent Calcium Carbonate in Soil  [percent \| 100m] | Mean percent calcium carbonate in the first 0-5cm of the soil horizon | [POLARIS](https://www.sciencedirect.com/science/article/pii/S0016706116301434)^7^ |  |
| Pore Size Distribution Index  [unitless \| 100m] | Mean pore size distribution index in the first 0-5cm of the soil horizon | [POLARIS](https://www.sciencedirect.com/science/article/pii/S0016706116301434)^7^ | High correlations and redundancy |
| Available Water Content (depth, cm)  [m^3^/m^2^ \| 100m] | Variance of available water content in the first 0-5cm of the soil horizon | [POLARIS](https://www.sciencedirect.com/science/article/pii/S0016706116301434)^7^ |  |
| Available Water Content (depth, cm)  [m^3^/m^3^ \| 100m] | Mean available water content in the first 0-5cm of the soil horizon | [POLARIS](https://www.sciencedirect.com/science/article/pii/S0016706116301434)^6^ |  |
| Soil Water Content at Field Capacity (0-5 cm)[m^3^/m^3^ \| 100m] | Mean soil water content at field capacity in the first 0-5cm of the soil horizon | [POLARIS](https://www.sciencedirect.com/science/article/pii/S0016706116301434)^7^ | Replaced with new dataset based on expert feedback |
| Depth to Restriction Layer  [cm \| 100m] | Mean depth to restriction layer | [POLARIS](https://www.sciencedirect.com/science/article/pii/S0016706116301434)^7^ |  |
| Distance to Water (coarse scale)  [meters \| 90m] | Euclidean distance to the nearest waterbody or flowline feature using coarse resolution water features | [National Atlas](https://www.arcgis.com/home/item.html?id=0eb5f7b586ea4e08b5003b3554032453)^8^ | Added to address INHABIT feedback and improve model sensitivity within riparian areas |
| Distance to Water (fine scale)  [meters \| 90m] | Euclidean distance to the nearest waterbody or flowline feature using high resolution water features | [NHDPlus](https://www.sciencebase.gov/catalog/item/56c38ad8e4b0946c6520aa52)^6^ | Added to address INHABIT feedback and improve model sensitivity within riparian areas |
| Bulk Density  [kg / m^3^ \| 100m] | Bulk density (fine earth) at 0.05m depth, or soil mass per volumetric unit | [Soil Properties](https://scholarsphere.psu.edu/resources/ea4b6c45-9eba-4b89-aba6-ff7246880fb1)^9^ | Substituted based on expert feedback |
| Percent Clay  [percent \| 100m] | Clay content (0-2 µm) mass fraction (percent) at 0.05m depth | [Soil Properties](https://scholarsphere.psu.edu/resources/ea4b6c45-9eba-4b89-aba6-ff7246880fb1)^9^ | Substituted based on expert feedback |
| Potassium Content  [ppm \| 100m] | Potassium content in ppm at 0.05m depth | [Soil Properties](https://scholarsphere.psu.edu/resources/ea4b6c45-9eba-4b89-aba6-ff7246880fb1)^9^ | Important for new plant growth and available through new dataset |
| Nitrogen Content  [100m] | Total (organic) Nitrogen content at 0.05m depth | [Soil Properties](https://scholarsphere.psu.edu/resources/ea4b6c45-9eba-4b89-aba6-ff7246880fb1)^9^ | Important for new plant growth and available through new dataset |
| Soil pH [100m] | Soil pH x 10 in H2O at 0.05m depth | [Soil Properties](https://scholarsphere.psu.edu/resources/ea4b6c45-9eba-4b89-aba6-ff7246880fb1)^9^ | Important for new plant growth and available through new dataset |
| Percent Sand  [percent \| 100m] | Sand content (50-2000 µm) mass fraction (percent) at 0.05m depth | [Soil Properties](https://scholarsphere.psu.edu/resources/ea4b6c45-9eba-4b89-aba6-ff7246880fb1)^9^ | Substituted based on expert feedback |
| Soil Organic Carbon  [g/kg \| 100m] | Soil organic carbon content (fine earth fraction) in g per kg at 0.05m depth | [Soil Properties](https://scholarsphere.psu.edu/resources/ea4b6c45-9eba-4b89-aba6-ff7246880fb1)^9^ | Important for new plant growth and available through new dataset |
| Remoteness (Night Lights)  [brightness \| 250m] | Nighttime lights of 2011. Cloud-free composites use visible and infrared sensors to capture imagery. | [NOAA](https://ngdc.noaa.gov/eog/dmsp/downloadV4composites.html)^10^ | Replaced with gHM |
| Land Surface Temperature (MODIS)  [0-254 \| 1,000m] | Land surface temperature/emissivity | [NASA](https://modis-land.gsfc.nasa.gov/temp.html) |  |
| Maximum Burn Severity  [0-6 \| 30m \| 1984 – 2015] | Maximum burn severity | [MTBS](https://www.mtbs.gov/product-descriptions)^11^ | High correlations and redundancy |
| Year of Last Fire  [1-32 \| 30m \| 1984 – 2015] | Year of the last fire | [MTBS](https://www.mtbs.gov/product-descriptions)^11^ | High correlations and redundancy |
| Severity of Last Fire  [0-6 \| 30m \| 1984 – 2015] | Burn severity of last fire | [MTBS](https://www.mtbs.gov/product-descriptions)^11^ | High correlations and redundancy |
| Burn Frequency  [1-32 \| 30m \| 1984 – 2015] | Burn frequency, or the number of times a particular area has burned over the given time period | [MTBS](https://www.mtbs.gov/product-descriptions)^11^ | Added additional years of fire data [1984 – 2018] |
| NDMI median  [-1 to 1 \| 30m \| 1984 – 2020] | Normalized Difference Moisture Index (NDMI) median between April 1st and September 30th spanning 1985 to 2020 derived from Landsat 5, 7, and 8 time series analysis. This is useful for summarizing growing season surface water which can affect the distribution of a species. | [Land-Trendr](https://emapr.github.io/LT-GEE/introduction.html) & Halmy et al., 2019^12^ | Added to address INHABIT feedback and improve model sensitivity within riparian areas |
| NDMI standard deviation  [-1 to 1 \| 30m \| 1984 – 2020] | Normalized Difference Moisture Index (NDMI) standard deviation between April 1st and September 30th spanning 1985 to 2020 derived from Landsat 5, 7, and 8 time series analysis. This is useful for summarizing the variability of growing season surface water which can affect the distribution of a species. | [Land-Trendr](https://emapr.github.io/LT-GEE/introduction.html) & Halmy et al., 2019^12^ | Added to address INHABIT feedback and improve model sensitivity within riparian areas |
| Water Recurrence  [0 -1200 \| 30m \| 1984 – 2019] | Annual water recurrence within a pixel. The average monthly percentage cover of water within a pixel (1984 - 2019), summed across all 12 months. This is useful for summarizing annual surface water which can affect species’ distribution of a species. | [Global Surface Water](https://developers.google.com/earth-engine/datasets/catalog/JRC_GSW1_2_MonthlyRecurrence#citations)^13^ | Added to address INHABIT feedback and improve model sensitivity within riparian areas |
| Bare Ground Standard Deviation  [percent stddev \| 250m \|2010 – 2016] | Standard deviation of percent cover of bare ground over the given time period | [NASA - LP DAAC](https://lpdaac.usgs.gov/products/mod44bv006/)^14^ |  |
| Mean Tree Cover  [percent \| 250m \| 2010 – 2016] | Mean tree cover over the given time period | [NASA - LP DAAC](https://lpdaac.usgs.gov/products/mod44bv006/)^14^ |  |
| CHILI  [1 to 254 \| 10m] | Continuous Heat-Load Index (CHILI) captures the effect of topographic shading and insolation on evapotranspiration values. It is calculated using USGS's NED DEM (10m). | [Theobald et al., 2015](https://journals.plos.org/plosone/article?id=10.1371/journal.pone.0143619)^15^ | Important for describing local variation in evapotranspiration. |
| mTPI  [-319 to 421 \| 270m] | Multi-Scale Topographic Position index (mTPI) is used to differentiate valleys and ridges and characterize the slope of the terrain. It is based off of USGS's NED DEM (10m) and calculated using elevation values minus the mean neighborhood elevation using variable radii (1.2, 2.8, 5.6, 13.1, 35.5, 89.9, and 115.8 km respectively). | [Theobald et al., 2015](https://journals.plos.org/plosone/article?id=10.1371/journal.pone.0143619)^15^ | Important for describing local topographic variability and characterizing landscape features. |
| Topographic Diversity  [0 to 1 \| 90m] | Topographic Diversity captures the variability in moisture and temperature conditions within local habitats, and it is calculated using a combination of mTPI and CHILI (see mTPI and CHILI for details). Areas with greater topographic and climatic diversity should support greater species diversity. | [Theobald et al., 2015](https://journals.plos.org/plosone/article?id=10.1371/journal.pone.0143619)^15^ | Important for describing the compounding effects that topographic variability has on evapotranspiration |

The variable column includes the name, listed within brackets are the unit of measurement, spatial (cell) resolution, and temporal resolution (if applicable). Each variable also has a specific description and source. Red variable names represent first version variables removed from the second version of the predictor list due to high correlations, redundancy, or contributing little importance to models. Yellow variable names represent second version variables that replaced a similar predictor from the first version. Green variable names represent second version variables that were created in response to feedback and added to the second version of the predictor list to improve model sensitivity. Variable names without an associated color represent first version variables that were retained in the second version list without being modified.

^1^ Daly, C., Halbleib, M., Smith, J.I., Gibson, W.P., Doggett, M.K., Taylor, G.H., Curtis, J., Pasteris, P.P. “Physiographically sensitive mapping of climatological temperature and precipitation across the conterminous United States”. International Journal of Climatology. (2008) 28, 2031–2064. <https://doi.org/10.1002/joc.1688>. AND

O’Donnell, M.S. and Ignizio, D.A. (2012). Bioclimatic predictors for supporting ecological applications in the conterminous United States. US Geological Survey Data Series, 691(10).

^2^Abatzoglou J. T. " Development of gridded surface meteorological data for ecological applications and modelling " International Journal of Climatology. (2011) doi: 10.1002/joc.3413.

^3^M. Kennedy, Christina; Oakleaf, James; M. Theobald, David; Baruch-Mordo, Sharon; Kiesecker, Joseph (2018): Global Human Modification. figshare. Dataset. https://doi.org/10.6084/m9.figshare.7283087.v1

^4^Hak, J.C. and P.J. Comer. 2017. Modeling Landscape Condition for Biodiversity Assessment – Application in Temperate North America. Ecological Indicators 82:206-216.

^5^Wildlife Conservation Society - WCS, and Center for International Earth Science Information Network - CIESIN - Columbia University. (2005). Last of the Wild Project, Version 2, 2005 (LWP-2): Global Human Influence Index (HII) Dataset (Geographic). Palisades, NY: NASA Socioeconomic Data and Applications Center (SEDAC). https://doi.org/10.7927/H4BP00QC.

^6^U.S. Environmental Protection Agency (USEPA) & U.S. Geological Survey (USGS). (2012). *National Hydrography Dataset Plus - NHDPlus - ScienceBase-Catalog* (2.1) [Dataset]. https://www.sciencebase.gov/catalog/item/56c38ad8e4b0946c6520aa52

^7^Chaney NW, Wood EF, McBratney AB, Hempel JW, Nauman TW, Brungard CW, Odgers NP. 2016. POLARIS: A 30-meter probabilistic soil series map of the contiguous United States. Geoderma 274: 54-67.

^8^ESRI, National Atlas of the United States, & U.S. Geological Survey (USGS). (2010). *USA Water Bodies, Rivers & Streams* [Dataset]. https://www.arcgis.com/home/item.html?id=0eb5f7b586ea4e08b5003b3554032453; https://www.arcgis.com/home/item.html?id=8206e517c2264bb39b4a0780462d5be1

^9^Nauman, T., Ramcharan, A., Brungard, C., Thompson, J., Wills, S., Waltman, S., & Hengl, T. (2017). *Soil Properties and Class 100m Grids United States* [Dataset]. PennState University Libraries. https://doi.org/10.18113/S1KW2H

^10^NOAA’s National Geophysical Data Center & US Air Force Weather Agency. (2011). *Version 4 DMSP-OLS Nighttime Lights Time Series* (Version 4) [Dataset]. Image and data processing by NOAA’s National Geophysical Data Center. DMSP data collected by US Air Force Weather Agency. https://ngdc.noaa.gov/eog/dmsp/downloadV4composites.html

^11^Eidenshink, J., B. Schwind, K. Brewer, Z. Zhu, B. Quayle, and S. Howard. (2007). A project for monitoring trends in burn severity. Fire Ecology 3(1): 3-21.

^12^Kennedy, R.E., Yang, Z., Gorelick, N., Braaten, J., Cavalcante, L., Cohen, W.B., Healey, S. (2018). Implementation of the LandTrendr Algorithm on Google Earth Engine. *Remote Sensing*. 10, 691. AND

Halmy, M. W. A., Fawzy, M., Ahmed, D. A., Saeed, N. M., & Awad, M. A. (2019). Monitoring and predicting the potential distribution of alien plant species in arid ecosystem using remotely-sensed data. Remote Sensing Applications: Society and Environment, 13, 69–84. https://doi.org/10.1016/j.rsase.2018.10.005

^13^Jean-Francois Pekel, Andrew Cottam, Noel Gorelick, Alan S. Belward, (2016). High-resolution mapping of global surface water and its long-term changes. *Nature* 540, 418-422. [doi:10.1038/nature20584](https://doi.org/10.1038/nature20584)

^14^DiMiceli, C. M., Carroll, M. L., Sohlberg, R. A., Huang, C., Hansen, M. C., & Townshend, J. R. G. (2011). Annual global automated MODIS vegetation continuous fields (MOD44B) at 250 m spatial resolution for data years beginning day 65, 2000– 2010, collection 5 percent tree cover. College Park, MD: University of Maryland.

^15^Theobald, D. M., Harrison-Atlas, D., Monahan, W. B., & Albano, C. M. (2015). Ecologically-relevant maps of landforms and physiographic diversity for climate adaptation planning. PloS one, 10(12), e0143619 https://doi.org/10.1371/journal.pone.0143619
