# Supplementary material for: INHABIT: A web-based decision support tool for invasive plant species habitat visualization and assessment across the contiguous United States
Source: PLoS One. 2022 Feb 8;17(2):e0263056. doi: 10.1371/journal.pone.0263056 (PMC8824347; doi:10.1371/journal.pone.0263056)
Supplement: Attachment — Submitted filename: Response to Reviewers.docx [file pone.0263056.s002.docx]

Dr. Silva,

Thank you for giving us the chance to submit a revised version of our manuscript. We are pleased that the reviewers valued the contribution provided by our manuscript, and appreciate the thoughtful comments and suggestions you and the reviewers were able to provide. As suggested by Reviewer #1, our manuscript now reflects important changes to more closely align with PLOS ONE’s criteria for “Methods, software, databases, and tools”. Namely, we have reworked the introduction to provide more detail on the methods currently used to generate invasive plant management actions and include more detail on the novelty of the tool in this context. We also now include a third subsection to the case studies that expands upon the ways in which INHABIT has and will change as a direct result of user feedback.

In addition to the above comments, figure two has been revised to remove any copyright content. Figures four and five were flagged for possible copyright violations but they both only contain image content from our model outputs. Minor comments have been incorporated, and we have edited for clarity where suggested. See below for detailed responses to both reviewers.

Comments for Reviewer #1

We appreciate the reviewer’s suggestion that, as a description of a real-world application, the content of this manuscript falls under the “Methods, software, databases, and tools” category. To better structure the manuscript to fit the criteria of these types of submissions (namely, *Utility* and *Validation*), the reviewer suggested the following:

*“provide more details on the proven advantage over existing alternatives “*

We have edited a significant portion of the introduction to emphasize the novelty of this tool and describe its difference from currently available tools. From conversations with land managers and practitioners, we are not aware of any available tool equivalent to INHABIT. Without INHABIT, occurrence locations alone shape much of the spatial aspect of invasive species management and assessment in the United States. The use of occurrence locations in isolation paints an incomplete picture of the landscape and so can be complemented and filled in by predictions of habitat suitability from species distribution models. Overall, our tool represents a fundamental shift in the accessibility, interpretability, and utility of species distribution model outputs for the explicit purpose of invasive species management.

*“potential for future growth? Perhaps there are insights from the case studies”*

*“the long term utility is implicit, but again, give more insights from the case studies, for example, any surveys from the users, or contracts with other users, or feedback submitted to the platform?”*

To address these closely related comments, we have added a third section to the case studies entitled “Iterating INHABIT through feedback”. In this new section, we go into detail regarding the changes (big and small) to INHABIT that have already been implemented as a direct result of user feedback. Also highlighted are upcoming changes and their anticipated impact on the invasive species management community at large. The changes to the website come from a variety of sources, including the feedback tab, ongoing partner conversations, and web analytics analysis. In the future, a more formal usability survey may be conducted to help determine specific enhancements to web-design elements of INHABIT.

*“the case studies do show validation”*

We agree with the reviewer that validation is an important part of our work. Collaboration from the National Park Service has been very fruitful in providing on-the-ground confirmation of the SDM predictions available through INHABIT. Continually evolving validation strategies are also anticipated for future model workflow iterations, with more robust testing and training splits building off the current methods from Young et al, 2020.

Comments for Reviewer #2

We thank the reviewer for their enthusiasm about our work and agree that INHABIT is a boon to management planning through ease of access, downloadable materials, and unique visualizations. We appreciate the reviewer’s recommendation for publication and that, while the edits mentioned above represent changes to the manuscript, the fundamental structure has not been altered significantly.

We look forward to hearing from you in due time regarding our submission and to respond to

any further questions and comments you may have.

Sincerely,

Peder Engelstad

[peder.engelstad@colostate.edu](mailto:peder.engelstad@colostate.edu)

+1 206-478-1474

9/21/2021
